# Penoscrotal Edema as a Rare Complication of Acute Pancreatitis: A Case Report

**DOI:** 10.3390/medicina60050820

**Published:** 2024-05-16

**Authors:** Ivana Jukic, Visnja Kokic Males, Antonija Zanic, Ivan Zaja

**Affiliations:** 1 Department of Gastroenterology and Hepatology, University Hospital Split, Spinciceva 1, 21000 Split, Croatia; ivjukic@gmail.com (I.J.); ivan.zaja@yahoo.com (I.Z.); 2 Department of Health Studies, University of Split, 21000 Split, Croatia; 3 Institute of Emergency Medicine of Split—Dalmatia County, 21000 Split, Croatia; zanicantonija@gmail.com

**Keywords:** acute pancreatitis, scrotal edema, hydrocele, swelling, peripancreatic fluid

## Abstract

*Background and Objectives*: Scrotal swelling or hydrocele is a rare complication of acute pancreatitis described in the literature. We present a case of penoscrotal swelling caused by the first attack of acute interstitial edematous alcohol-induced pancreatitis in a young male patient. *Case report*: A 22-year-old man was admitted to the emergency unit due to diarrhea and vomiting since morning which was followed by severe abdominal pain. Urgent abdominal multislice CT scan showed steatosis, pancreatic swelling and acute peripancreatic fluid collection (interstitial edematous pancreatitis). Also, scan showed fluid between small bowel loops and along the anterior renal fascia, while there was minimal amount of fluid in the Douglas space. There was no sign of penoscrotal swelling. On the second day of admission, the patient developed left scrotal swelling and mild pain without erythema. On the fourth day, a control CT scan showed progression to moderately severe pancreatitis (CT severity index 4). Dilated scrotal veins of the pampiniform venous plexus with an increased caliber of the testicular veins were present on both sides, from the scrotum to the level of the inguinal canal. Penoscrotal swelling was significantly reduced on discharge. *Conclusions*: Penoscrotal swelling is a rare complication or manifestation of acute inflammation of the pancreas. It is important to identify scrotal swelling caused by pancreatitis because in severe cases it can be related to possible infertility in the future.

## 1. Introduction

Acute pancreatitis is one of the most common pathologies of the gastrointestinal tract, leading to tremendous emotional, physical, and financial burden. Acute pancreatitis requires urgent hospitalization, and patients often develop additional complications that can be local or systemic. Local inflammation of the gland and the cytokines that are released into the circulation contribute to the characteristic symptoms of acute pancreatitis. The annual incidence rate is about 13–45 cases per 100,000 adults, and the overall mortality is around 5% [1]. In interstitial (edematous) pancreatitis, the mortality is < 5%, while in necrotizing pancreatitis mortality rates are significantly higher. The most common triggers of AP are gallstones and chronic heavy alcohol intake. Less common causes include hypertriglyceridemia [1], trauma, pancreatic or periampullary cancer, autoimmune diseases, malfunction of the parathyroid gland, infections, congenital pancreaticobiliary anomalies, such as pancreas divisum and stenosis of the sphincter of Oddi, idiopathic causes, and drugs [1]. Local complications are defined as peripancreatic fluid collections, pancreatic and peripancreatic necrosis (sterile or infected), pseudocysts, and walled-off necrosis (sterile or infected). Local fluid collections can also lead to ascites, pleural effusion, and acute respiratory distress syndrome [1].

However, scrotal swelling or hydrocele is a rare presentation of acute pancreatitis described in the literature so far [2,3,4,5,6,7,8,9,10,11,12,13,14,15]. According to previous study, 57% of patients with acute pancreatitis were diagnosed to have fluid collections, 39% having two and 33% having three or more areas involved [16]. The extension of a pancreatic collection, pseudocyst, or abscess into the groin, involving the inguinal canal and scrotum is a rare complication of acute pancreatitis. In spite of the low incidence of this complication, it does capture our attention as it may be misdiagnosed as other more common pathologies of scrotum swelling such as orchiepididymitis, testicular torsion, testicular hydrocele, or testicular tumor, and may lead to unnecessary surgical treatment [12,17,18].

We present a rare case of penoscrotal swelling caused by the first attack of acute interstitial alcohol-induced pancreatitis.

### Case Presentation

**Figure 1:**
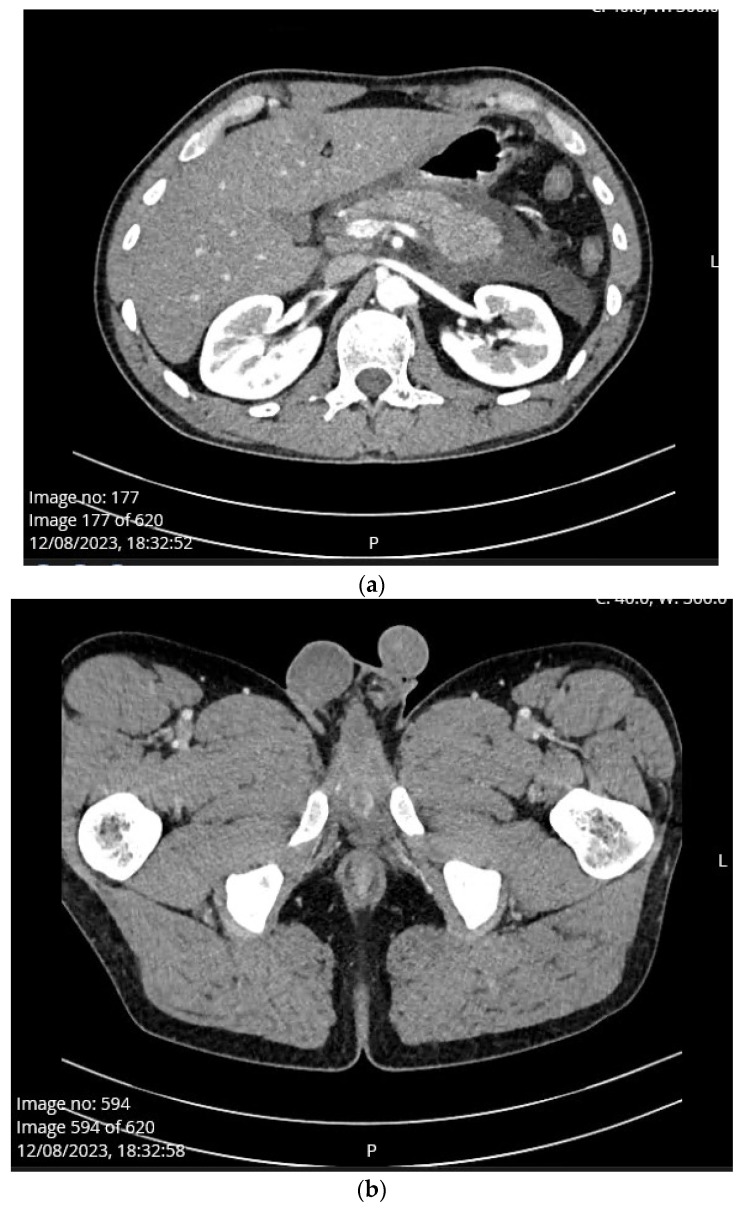
(**a**). Axial contrast abdominal CT scan showed fatty liver disease, pancreatic swelling, and acute peripancreatic fluid collections (interstitial edematous pancreatitis), fluid between the intestinal coils and along the anterior renal fascia, and minimal fluid in the Douglas space on the first day of admission. (**b**). Axial contrast CT scan showed there was no sign of penoscrotal swelling on the first day of admission.

**Figure 2:**
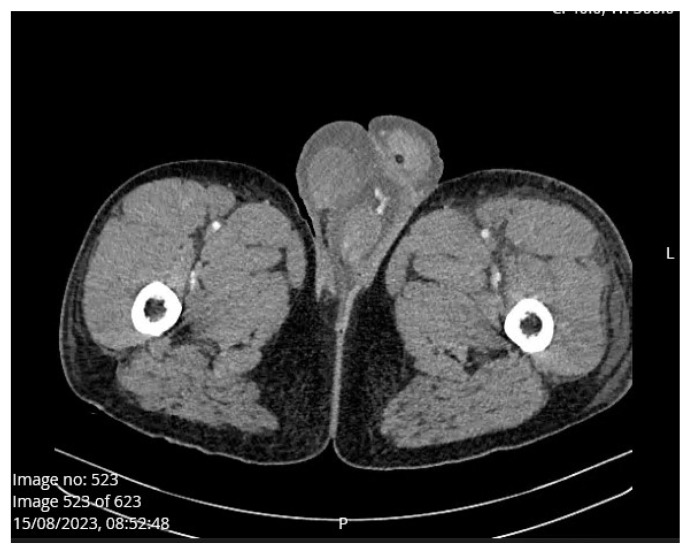
Axial contrast CT scan showed dilated scrotal veins of the pampiniform venous plexus with the indicated caliber of the testicular veins on both sides from the scrotum to the level of the inguinal canal—a progression compared to the earlier finding. There was no sign of acute compartment syndrome. The amount of free fluid in the scrotum and subcutaneous tissue of the penis was also progressing—edema. The testicles, spongy and cavernous bodies of the penis are properly imbibed post-contrast. Bilateral inguinal subcutis edema.

A 22-year-old man was admitted to the Emergency Unit of Clinical Hospital Centre Split, due to diarrhea as the first clinical sign present since morning followed by vomiting and severe abdominal pain. No associated fever and chills, urinary, trauma, or procedure history was noted. He reported excessive alcohol drinking and eating grilled meat the night before pain onset. He was previously healthy. Upon admission to the hospital, physical examination revealed tense and extremely painful abdominal wall to palpation; however, there were no signs of tender testis, epididymis, or palpable mass at inguinal region. Blood pressure was 151/110 mmHg and a physical exam revealed no other pathological signs. The patient was a smoker and consumed alcoholic beverages frequently. Urgent abdominal multislice computed tomography scan (Figure 1a) showed steatosis, pancreatic swelling, and acute peripancreatic fluid collections which was consistent with interstitial edematous pancreatitis. Also, there was a significant amount of fluid between the small bowel loops and along the anterior renal fascia. There was minimal amount of fluid in the Douglas space. There was no sign of penoscrotal swelling. (Figure 1b). Initial laboratory workup revealed a serum lipase level of 853 U/L (13–60 U/L), CRP level of 128.1 mg/L (0–5 mg/L), procalcitonin level of 1.06 ng/mL (0–0.05 ng/mL), and lactate level of 3.5 mmol/L (0.5–2.2 mmol/L). The patient was admitted to the Intensive Care Unit of Department of Gastroenterology and an APACHE II score was determined at the end of the first 24 h of ICU admission. The APACHE II score was 5, while the BISAP score was 0. A chest X-ray was within normal range. On the second day of admission, the patient developed left scrotal swelling and mild pain without erythema. Even though the urology resident suspected a left varicocele, a senior consultant urologist did not reveal any abnormal physical status of either testis or epididymis. MSCT scan on admission and after developing penoscrotal edema did not confirm varicocele (Figure 1b and Figure 2). Axial contrast CT scan showed the dilated scrotal veins of the pampiniform venous plexus with the indicated caliber of the testicular veins on both sides, from the scrotum to the level of the inguinal canal, had progressed compared to the earlier finding. There was no sign of acute compartment syndrome. The amount of free fluid in the scrotum and subcutaneous tissue of the penis was also progressing to bilateral inguinal subcutis edema (Figure 2).

**Figure 3:**
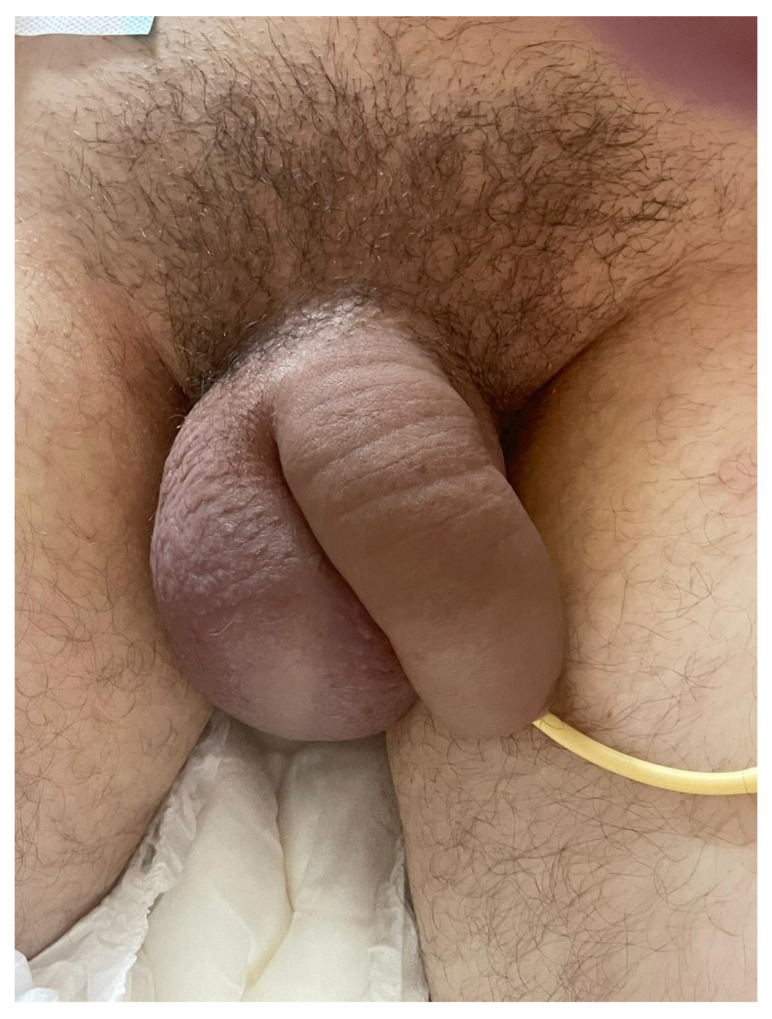
Penoscrotal swelling in 22-year-old patient.

On the second day of admission laboratory workup revealed serum lipase level of 377 U/L, CRP level of 286.1 mg/L, serum protein level of 36 g/L (66–91 g/L) and albumin level of 19.4 g/L (40.2–47.6 g/L). Regarding the progression of edema of both scrotum and penis (Figure 2), a control MSCT scan was performed on the fourth day of admission (Figure 3). There were newly formed pleural effusions on both sides, up to 4.2 cm wide on the right, and up to 3.8 cm on the left. The pancreas was edematous, without obvious zones of necrotic tissue. There was significant peripancreatic, perisplenic, perihepatic, and pelvic floor progression of the amount of free fluid which corresponds to the CT criteria of moderately severe pancreatitis, which was in progression compared with previous findings (CT severity index- CTSI 4). Small and large intestine loops were normally calibrated. There was no free gas in the peritoneum. There was also some edema in the root of the mesentery. Dilated scrotal veins of the pampiniform venous plexus with increased caliber of the testicular veins were present on both sides, from the scrotum to the level of the inguinal canal. There was no sign of acute compartment syndrome. The amount of free fluid in the scrotum and subcutaneous tissue of the penis was also progressing. The testicles, spongy and cavernous bodies of the penis were properly imbibed with contrast. Bilateral inguinal subcutis edema was present. Intra-abdominal pressure was 18 cm of H_2_0 (intra-abdominal hypertension grade II). There was no sign of acute compartment syndrome during hospitalization. The serum levels of alfa fetoprotein and β-hexachlorocyclohexane (β-HCH) were within normal range. During hospitalization the patient was treated with nasogastric suction, meropenem, metronidazol, pantoprazole, enoxaparin, furosemide, 20% humane albumin, electrolyte infusion, analgesic therapy, paracetamol, parenteral and enteral nutrition, and dietary modifications. Although retrograde endoscopic cholangiopancreatography (ERCP) can be a therapeutic option in pancreatitis, but also lead to post-ERCP pancreatitis [19], in our case report, ERCP was not indicated regarding the etiology of the disease. Imaging methods did not establish biliary pancreatitis (due to choledocholithiasis), but alcohol-induced acute pancreatitis. Pharmacological therapy was the therapy of choice, and surgical treatment was not necessary since acute compartment syndrome was ruled out. There was no specific treatment for penoscrotal edema, but treatment of acute pancreatitis led to spontaneous remission of penoscrotal edema. After 13 days of hospitalization, he was discharged due to significant clinical improvement, with laboratory tests within normal range. The following control tests are recommended: ultrasound of the scrotum, CT abdominal scan, chest X-ray, reproductive hormone levels, sperm and semen analysis. Unfortunately, we wanted to perform sperm and semen analysis, but our patient refused to perform this diagnostic procedure for unknown reasons, and he was not citizen of Republic of Croatia and outside our jurisdiction. Penoscrotal swelling was significantly reduced on discharge.

## 2. Discussion

In this article, we presented a rare case of penoscrotal swelling caused by the first attack of acute interstitial alcohol-induced pancreatitis in a young male patient. According to Moens L. et al. until 2016, 18 cases of inguinoscrotal swelling in acute pancreatitis have been described [12]. In most cases (*n* = 11), pancreatitis was alcohol induced, 14 cases had interstitial edematous acute pancreatitis, and 4 cases of necrotizing pancreatitis. By searching the PubMed database, 26 cases of swelling of the penoscrotal and inguinal region in patients with acute pancreatitis have been published [2,3,4,5,6,7,8,9,10,11,12,13,14,15]. Previous studies proposed that hydrocele is a result of peripancreatic fluid in the retroperitoneum tracking through pelvic space and inguinal canal into the scrotum along processus vaginalis [7,8,9,15]. The patients had failure of closure of the processus vaginalis, so consequently the fluid dissected between the visceral and parietal layers of the tunica vaginalis led to communicating hydrocele. Previous authors have noticed that pancreatic swelling was rare manifestation of mostly alcohol-related pancreatitis among different etiologies of pancreatitis [7,12,13,15]. Alcohol may increase the production of digestive and lysosomal enzymes and result in more fluid collections. In the last published case, however, retroperitoneal fluid was scanty, which was different from common causes of pancreatic hydrocele. No strong evidence revealed a direct link between scrotal effusion and retroperitoneal fluid. Instead, blockage of right testicular vein was noted. Chang et al. suggested the effusion of hydrocele, in their case, mainly resulted from venous congestion due to high pressure [2]. In this article we present a case of penis, bilateral scrotal, and inguinal region swelling caused by the first attack of acute interstitial alcohol-induced pancreatitis in a young previously healthy male patient. Measured intra-abdominal pressure was 18 cm H_2_O (intra-abdominal hypertension grade II). In general, intra-abdominal hypertension (IAH) is defined by pressures > 12 cmH_2_O (may be sufficient to restrict perfusion to the organs of the gut). IAH develops in 30% to 60% and acute compartment syndrome (ACS) in 15% to 30% of all acute pancreatitis patients and they are markers of severe disease with high morbidity and mortality. The detrimental effect of increased IAP has been recognized in several organ systems, including the central nervous system, cardiovascular, respiratory, renal, and gastrointestinal systems. The pathophysiology of IAH/ACS development in patients with acute pancreatitis is multifactorial. Pathogenetic mechanisms include over-zealous fluid management, visceral edema, ileus, peripancreatic fluid collections, ascites, and retroperitoneal edema (1). During hospitalization, the serum albumin levels (19.7 g/L, normal range 40.2–47.6 g/L) in our patient were very decreased, so a possible explanation for this penoscrotal swelling is generalized edema. We cannot exclude the previous possible explanation that peripancreatic fluid in the retroperitoneum extended down through pelvic space and inguinal canal into the scrotum along processus vaginalis. Hydrocele due to pancreatitis, usually subsides spontaneously once pancreatitis resolves under conservative treatment [5,11]. However, hydrocele may be attributed to congestive varicose vein of the testis that increases the risk of male infertility, so treating varicose vein is mandatory for severe cases. Physicians in clinical practice must know that penoscrotal edema can be one of the possible complications of acute pancreatitis. Differential diagnosis of penoscrotal edema can be misdiagnosed with varicocele, orchiepididmitis, testicular torsion, or even testicular tumor, which can often lead to wrong diagnosis and treatment.

The possible mechanisms of penoscrotal edema due to acute pancreatitis are described in Table 1. In the future, additional research is needed on a larger number of patients with penoscrotal edema in acute pancreatitis, in order to determine the exact pathophysiological mechanism of its occurrence.

## 3. Conclusions

In summary, we presented a case of penis, scrotal, and inguinal region swelling caused by the first attack of acute interstitial alcohol-induced pancreatitis in a young male patient. This is a rare complication/manifestation of acute pancreatitis, but it is important to identify scrotal swelling due to pancreatitis because it can be related to infertility. Until now, we can conclude hydrocele caused by acute pancreatitis should be carefully examined when evaluating local complications of acute pancreatitis. However, the sure pathophysiological mechanism of scrotoinguinal swelling remains partly unclear, as do the consequences for fertility. Additional research is needed on a larger number of patients in order to accurately determine the pathophysiological causes of penoscrotal edema, as well as its impact on fertility.

## Figures and Tables

**Table 1:** Possible mechanisms of penoscrotal edema in acute panreatitis.

Mechanisms of Penoscrotal Edema in Acute Panreatitis	Reference, Authors
1. Failure of closure of the processus vaginalis	1. Loloi, J. et al., 2019 [7]; Moens, L. et al., 2016 [8], Zafar et al., 2016 [9], Choong, K.K. 1996 [15]
2. Increased production of digestive and lysosomal enzymes due to alcohol	2. Loloi, J. et al., 2019 [7], Nikiforov, I. et al., 2015 [12], Wilde, C. et al., [13], Choong, K.K. 1996 [15]
3. Intra-abdominal hypertension (>12 cmH_2_O)	3. Aydin, H. et al., 2014. [19]
4. Hypoalbuminaemia	4. Ocskay, K. et al., 2021. [20]

## Data Availability

All data generated or analyzed during this study are included in this article. Further enquiries can be directed to the corresponding author.
